# An efficiency strategy for cobalt recovery from simulated wastewater by biphasic system with polyethylene glycol and ammonium sulfate

**DOI:** 10.1038/s41598-022-21418-7

**Published:** 2022-10-15

**Authors:** Razieh Sobhi Amjad, Mehdi Asadollahzadeh, Rezvan Torkaman, Meisam Torab-Mostaedi

**Affiliations:** 1 Faculty of Chemistry, Bu-Ali-Sina University, Hamedan, 65178-38683 Islamic Republic of Iran; 2 Nuclear Fuel Cycle Research School, Nuclear Science and Technology Research Institute, P.O. Box: 11365-8486, Tehran, Islamic Republic of Iran

**Keywords:** Engineering, Chemical engineering

## Abstract

Today, biphasic aqueous systems have received more attention than conventional separation methods due to their advantages, such as biocompatibility, low cost, and easy operation. The extraction of cobalt ions from the aqueous phase with the absence and the presence of other ions was investigated using polyethylene glycol, and ammonium sulfate salt without using an extractant. The efficiency was evaluated using operating parameters such as aqueous pH, salt and polymer concentrations, phase volume ratio, and initial metal concentration. The higher temperature, and the lower aqueous pH showed a maximum transfer rate for cobalt ions into the PEG1000 phase. Extraction efficiency under optimal conditions equal to 50% (w/w) polyethylene glycol 1000, 4 M ammonium sulfate, aqueous pH = 2, and 15 min extraction time was over 98%. Results from infrared spectroscopy, and thermo-gravimetric analysis illustrated the presence of the PEG-cobalt ion complex. The observation demonstrated that the biphasic system is the proper technology for wastewater purification.

## Introduction

Cobalt is heavy metal combined with other elements such as nickel, iron, and copper in the solar atmosphere, meteorites, and plants, among other places^1,2^. This metal is used to make corrosion-resistant alloys, ceramics, glass, paints and varnishes, and metal plating^3^. But the primary use of cobalt is in producing lithium and metal-nickel hydride batteries^4–6^. These batteries are primarily used in making mobile phones, tablets, laptops, and other devices. Lithium batteries are preferred to nickel-metal hydride batteries due to their low weight, size, and high voltage^7^. The widespread use of electronic devices containing lithium-ion batteries has produced large spent lithium-ion batteries in recent years. These batteries contain large amounts of heavy metals such as cobalt and harmful substances^8^.

Recycling cobalt from lithium-ion batteries is due to its low resources and widespread use of cobalt in the military and pharmaceutical industries, both environmentally friendly and economical^9,10^. Consequently, removing heavy metals from effluents is desirable for environmental reasons. In recent years, the isolation and recovery of cobalt from e-waste have been studied due to its widespread use in various industries.

Various methods have been suggested for the extraction and separation of metal ions. These methods include solvent extraction^11–13^, precipitation^14,15^, ion exchange^16^, surface and biological adsorption^17^, and membrane^18^ methods. Solvent extraction is a suitable method for metal separation and recycling. However, this extraction technique has disadvantages, such as using volatile and flammable organic solvents. As a result, developing environmentally friendly and safe extraction methods is necessary^19^.

An aqueous two-phase system (ATPS) is a new approach to extraction that can replace conventional extraction methods because they use water instead of toxic and flammable organic solvents^20,21^. Aqueous two-phase systems are usually used for separating and recovering biological products, metal ions, and dyes because of their low risk of causing less pollution^22^. The aqueous two-phase system has many benefits over other extraction processes, including non-flammability and non-toxicity, as well as biocompatibility and low material costs^23–25^. Aqueous two-phase systems involve mixing two incompatible polymers (PEG and dextran) in water or a water-soluble polymer and an inorganic salt (Na_2_SO_4_, (NH_4_)_2_SO_4_, Na_2_CO_3_)^26–28^. Therefore, the upper phase is rich in PEG, and the lower phase is rich in inorganic salt^29^. Because of its lower cost, lower viscosity, and high selectivity, the polymer/salt-based two-phase aqueous system is more widely employed^30–32^.

The partitioning mechanism of metal ions in an aqueous two-phase system must be fully understood further to clarify the physicochemical nature of the ionic partition process. It leads to new insights for improving the separation procedure. The main subject of earlier research works about the partition mechanism is related to the driving force of metal ions into the aqueous two-phase system. The electrostatic interaction theory has gained widespread acceptance in the past few decades as a valid explanation for how ions partition. Adding various complexing agents could significantly enhance the partitioning of metal cations. It was assumed that the metal cations would react with those complexing agents to create negatively charged species, which would then interact electrostatically with the protonated polyethylene glycol molecules^33,34^. In the study of Sun and co-workers, the partitioning of ions in the ATPS was first thought to be significantly influenced by the hydration characteristics of ions and their interface propensity^35^. In another study, due to the relatively high hydrophobicity of the polymolybdate anion and the salting-out effect of the phase-forming sodium sulfate, molybdenum was extracted into the PEG-rich phase by electrostatic attraction^36^.

The separation of aqueous two-phase systems depends on the molecular weight of the polyethylene glycol and the type of salt used. The aqueous two-phase system is easily formed by increasing the molecular weight of the polymer^37^. Increasing the molecular weight of the polymer reduces the hydrogen bond between the polyethylene glycol chains, which causes the metal ion to accumulate in the salt-rich phase. Therefore, polyethylene glycols with a molecular weight of 1000–8000 are used to extract metal ions. Increasing the salt concentration reduces the volume of the salt phase and causes the metal ions to move towards the polymer phase^38–40^.

Chung and co-workers showed that thallium could be extracted using a two-phase system of PEG and ammonium sulfate. It was found that the percentage of thallium extraction is affected by chlorine concentration^41^. Silva and co-workers studied the extraction of cobalt, nickel, cadmium, and iron by a two-phase aqueous system including tri-block copolymer and ammonium sulfate, polyethylene oxide, and lithium sulfate^42^. Azimate and co-workers studied the impact of temperature on a two-phase system of magnesium sulfate and polyethylene glycol (1500) at 35, 40, and 45 °C^43^. Bulgariu investigated the extraction of mercury at different halide concentrations. The maximum extraction is obtained at low concentrations of halide ions^29^. Extraction and removal of metal ions from industrial wastewaters due to their environmental friendliness and high separation efficiency were investigated using PEG (2000) and NaCrO_4_.H_2_O. The hydrophobic interaction plays a vital role in transferring chromium ions to the PEG-rich phase^35^.

A limited number of investigations are devoted to the extraction of cobalt ions with biphasic systems. In this study, the extraction of cobalt in a two-phase system of PEG and ammonium sulfate was investigated without using an extracting agent. The effects of various parameters such as salt concentration, PEG concentration, extraction time was investigated on the extraction percentage in the absence and the presence of other ions.

## Experimental

### Materials

Polyethylene glycol with molecular weights of 400 g/mol (pure liquid, CAS 25322–68-3), 1000 g/mol (pure solid, CAS 25322-68-3), and 10,000 g/mol (pure solid, CAS 25322-68-3) was purchased from Merck Company. Ammonium sulfate ((NH_4_)_2_SO_4_, ≥ 99.0%, CAS 7783–20-2), potassium chloride (KCl,  ≥ 99.0%, CAS 7447-40-7), potassium iodate (KIO_3_, 99.5%, CAS 7758-05-6), sodium chloride (NaCl,  ≥ 99.0%, CAS 7647-14-5), cobalt (II) sulfate (CoCO_4_.7H_2_O,  ≥ 99.0%, CAS 10026-24-1) were used in the experiments. They were prepared from Sigma Aldrich Company. The concentrated hydrochloric acid (fuming 37%, CAS 7647-1-0, Merck Company) and ammonia solution (25%, CAS 1336-21-6, Merck Company) were used to adjust the pH of an aqueous solution. The pH was adjusted by the addition of 1 M HCl solution or 1 M ammonia solution to the aqueous phase. It was measured using a Metrohm 691 pH meter, calibrated daily with standard buffer solutions. The deionized water was used to prepare the solutions. The other chemicals are reagents of analytical grade. All substances were utilized without further purification.

### Preparation of stock solutions

The stock solution of polyethylene glycol was obtained by dissolving different concentrations of polyethylene glycol in deionized water. A stock solution of ammonium sulfate salt was formed by dissolving some ammonium sulfate salt in deionized water and adjusting the pH with concentrated hydrochloric acid. Finally, a 500 ppm cobalt ion solution was generated by dissolving a specific amount of cobalt sulfate salt in deionized water. The leach sulfuric solution from zinc plant residue (Zanjan, Iran) was used in the experiments with the presence of other ions such as zinc, iron, nickel, and aluminum ions.

### Procedure

In this extraction, a two-phase aqueous system was obtained from a mixture of 3 ml of polyethylene glycol (50% w/w) in deionized water and 3 ml of ammonium sulfate solution (4 M) and 0.5 ml of metal ion solution (500 ppm Co(II) in pure system) in a 15 ml test tube. The resulting mixture was shaken for 30 min in a Memmert shaker at constant condition (T = 25 °C), and a 6000 rpm centrifuge was used for 10 min to separate the phases. All experiments were carried out at a fixed contact time of 30 min, based on the results of the preliminary experiments indicating that 30 min was sufficient to achieve equilibrium. The effect of phase ratio (PEG solution/(NH_4_)_2_SO_4_ solution), pH of saline solution, cobalt ion concentration, PEG and ammonium sulfate concentrations, and temperature was changed in the experiments to reach the maximum extraction efficiency.

**Figure 1:**
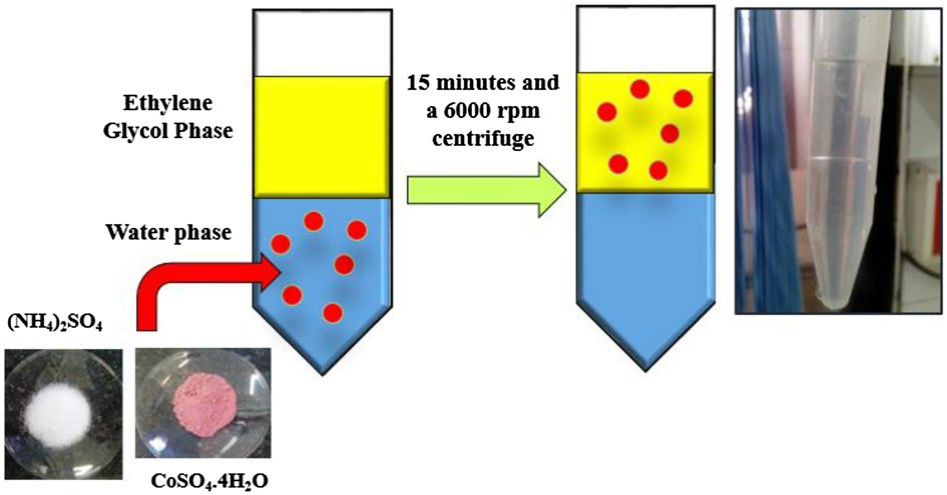
Schematic of procedure for cobalt extraction with an aqueous two-phase system.

After the two-phase system reached equilibrium, the phases were carefully separated by a pipette. The concentration of metal ions in the lower phase was determined by a UV spectrophotometer (Hach DR6000). Polyethylene glycol-cobalt (PEG-Co) coordination polymers have been characterized by FTIR spectroscopy (Bruker, victor22) and Thermogravimetric analyzer (TGA, STA 1500; Reoumetick scientific). The schematic of the procedure is shown in Fig. 1.

### Assessment of extraction efficiency

The following equation can be used to calculate the percentage of cobalt extraction:

1 $$ \% E = \frac{{(C)_{tp} }}{{(C)_{in} }} \times 100 $$

where (C)_tp_ is the concentration of the metal in the top phase and (C)_in_ is the concentration in the initial mass of the component in the feed solution.

The distribution coefficient has been used to evaluate the extraction efficiency.

2 $$ D = \frac{{(C)_{tp} }}{{(C)_{bp} }} $$

where (C)_tp_, and (C)_bp_ is the concentration of the metal in the top, and bottom phases, respectively. The selectivity (β) was obtained by the following equation:

3 $$ \beta = \frac{{D_{Co} }}{{D_{{other\begin{array}{*{20}c} {} \\ \end{array} ions}} }} $$

The experiments were repeated three times. The data and error bars indicate the average and standard deviations of three repeated determinations.

## Results and discussion

The selection of the appropriate two-phase-aqueous system has a significant impact on the extraction efficiency of a two-phase system. Consequently, selecting the type of constituent compound is the most crucial step in this extraction process. The effect of variables such as salt type and amount, polyethylene glycol type and amount, extraction time, phase ratio, and pH on extraction efficiency was investigated.

### Effect of PEG concentration and molecular weight

Polyethylene glycol with molecular weights of 400, 1000, and 10,000 was used to determine the appropriate molecular weight of the polymer. Table 1 depicts the effect of different molecular weights of polyethylene glycol on cobalt extraction in an ammonium sulfate salt medium. Low molecular weight polyethylene glycol requires high polymer or salt concentrations, according to the results^24^. The extraction percentage is reduced when the molecular weight of polyethylene glycol is increased from 1000 to 10,000. This decline in extraction percentage may be attributed to an increase in polyethylene glycol molecular chain length, which causes the hydroxyl groups of polyethylene glycol to decrease and the hydrophobic effect of polyethylene glycol to increase as the chain length increases. As a result, polyethylene glycol 1000 was chosen for further research based on the obtained results.

**Table 1:** Effect of PEG on the extraction of Co(II) (T = 25 °C; Time = 15 min; Co(II) = 500 ppm, PEG = 50% w/w, pH = 2).

Type	Hydrodynamic radius (nm)	Density (kg/m^3^)	Viscosity (mPa.s)	Extraction efficiency (%)
PEG 400	0.65	1061.5	1.400	85.56 ± 0.23
PEG 1000	0.93	1062.3	2.564	94.59 ± 0.17
PEG 10,000	2.29	1064.0	2.564	86.51 ± 0.19

**Figure 2:**
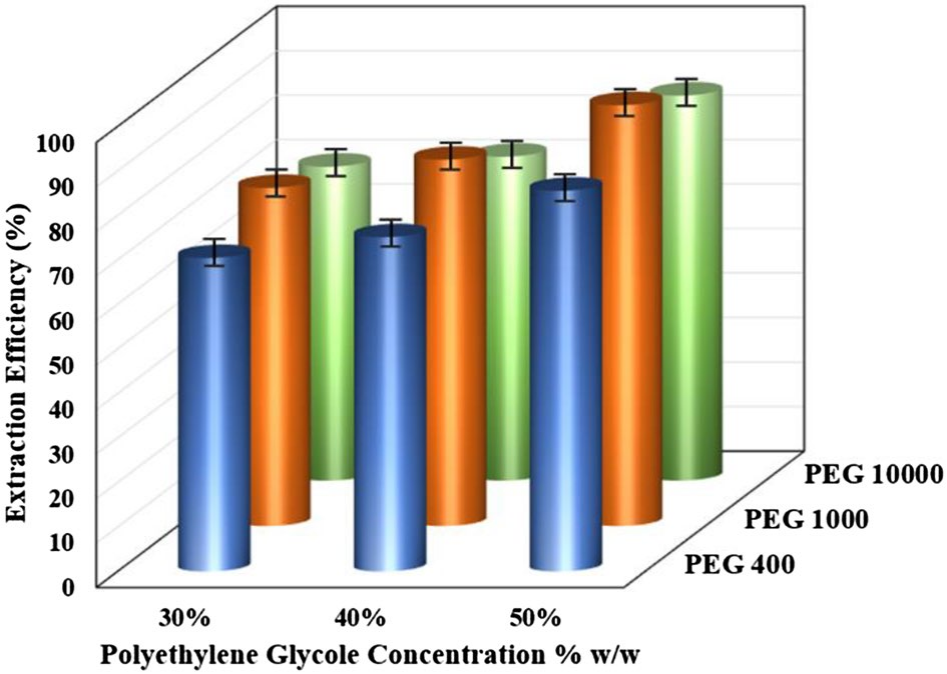
Effect of amount of PEG on the extraction of Co(II) (T = 25 °C, Time = 30 min, Co(II) = 500 ppm, (NH_4_)_2_SO_4_ = 4 M, pH = 2).

The results of the effect of the concentration of polyethylene glycol are presented in Fig. 2. According to the obtained results, increasing the concentration of polyethylene glycol increases the extraction performance (70.43, 75.11, 85.56% for PEG400 in 30%, 40%, and 50% concentration; 75.96, 79.55, 94.59% for PEG1000 in 30%, 40%, and 50% concentration; 70.51, 72.77, 86.51% for PEG10000 in 30%, 40%, and 50% concentration). It could increase in surface tension and viscosity between the phases of the two-phase system^44^. Furthermore, this system does not form two phases at concentrations less than 30% (w/w) of polyethylene glycol and ammonium sulfate.

### Effect of (NH_4_)_2_SO_4_ concentration

**Figure 3:**
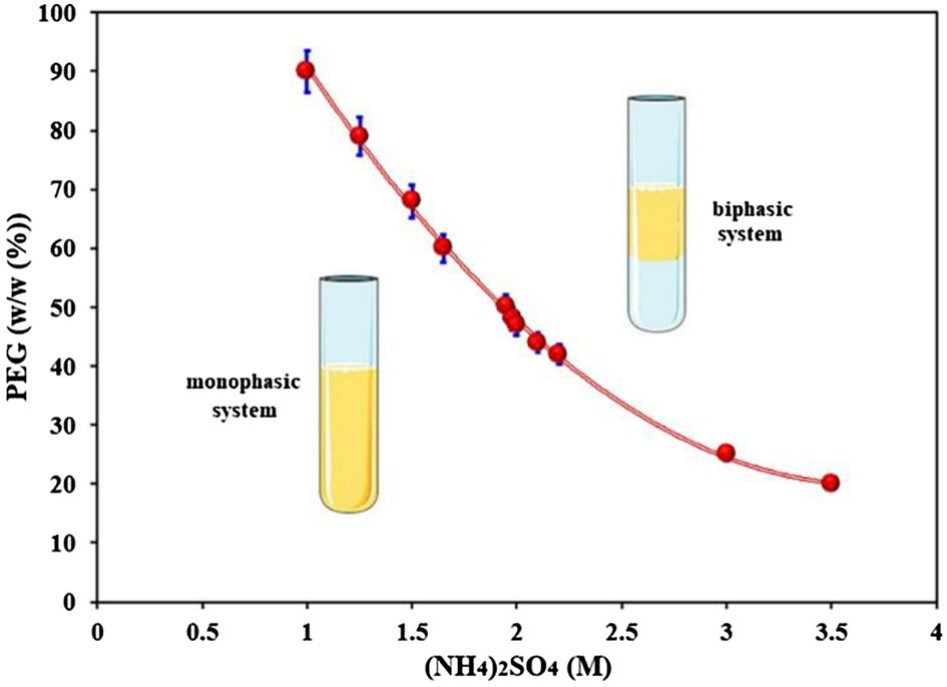
Phase diagram of (NH_4_)_2_SO_4_-polyethylene glycol (PEG) system for cobalt extraction (T = 25 °C, Time = 30 min, Co(II) = 500 ppm, pH = 2).

The binodal curve was investigated to describe the relationship between the concentration of (NH_4_)_2_SO_4_ solution and PEG solution with the presence of cobalt ions (see Fig. 3). The solution cannot shape two separate layers and exists as a monophase arrangement underneath the binodal bend. If the two polymer fixations are over the binodal bend, the aqueous solution isolates into layers of two phases, like water and oil.

**Figure 4:**
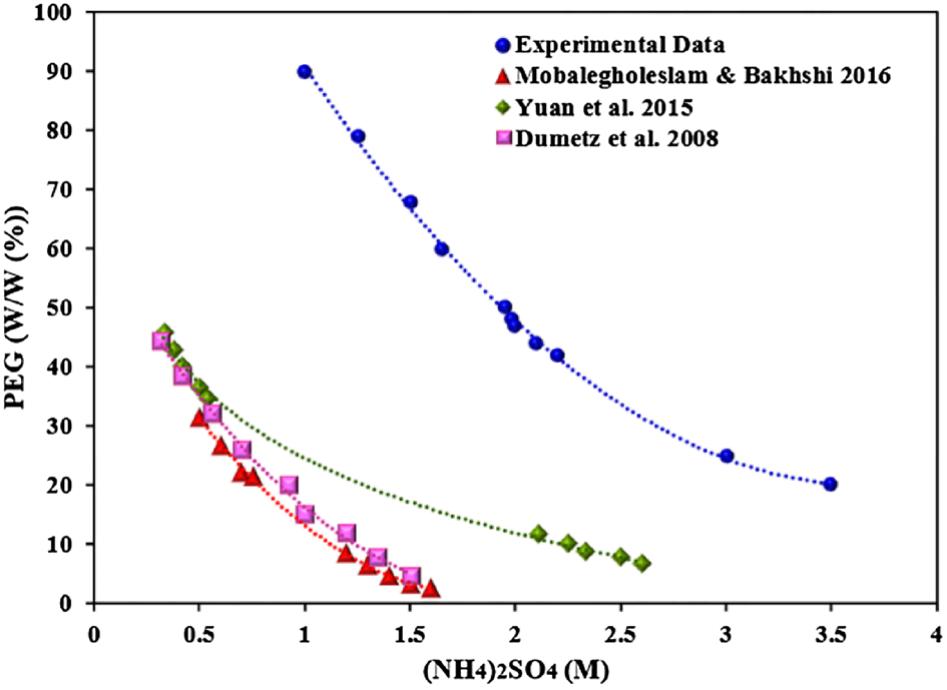
Comparison of the phase diagram of (NH_4_)_2_SO_4_-polyethylene glycol (PEG) system with the previous data in the literature.

The comparison of the obtained data in Fig. 3 with the results of Yuan and co-workers^45^, Mobalegholeslam and Bakhshi^46^ in the aqueous two phase system (ammonium sulfate and PEG) without cobalt ions and with protein reported by Dumetz and co-workers^47^ is shown in Fig. 4.

The results showed that the aqueous two-phase system containing cobalt ions was formed in higher concentrations of ammonium sulfate and polyethylene glycol. The aqueous two-phase curve has shifted to the right section. This state change in the protein extraction system is also formed in lower concentrations than cobalt ion extraction. Therefore, the type of extractive system and the hydration of polyethylene glycol at the interface is the main factor in the formation of an aqueous two-phase system.

**Figure 5:**
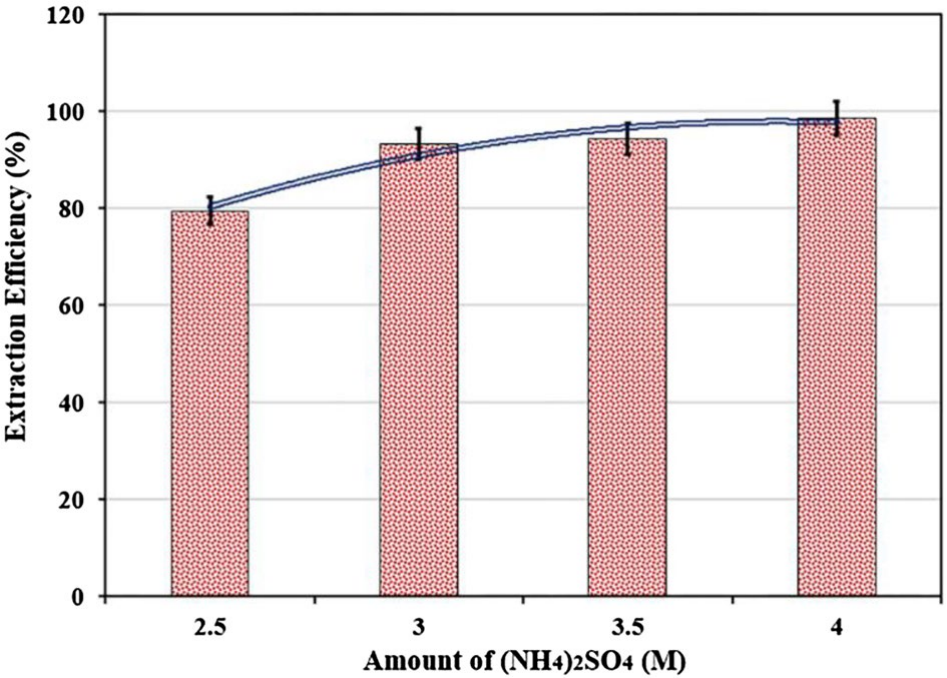
Effect of amount of (NH4)_2_SO_4_ on the extraction of Co(II) (T = 25 °C, Time = 30 min, Co(II) = 500 ppm, PEG = 50% w/w, pH = 2).

The effect of various concentrations of ammonium sulfate salt on cobalt extraction was studied, with the results shown in Fig. 5. According to the findings, the extraction percentage increases as the concentration of ammonium sulfate salt increases. This rise may be due to hydration rivalry between the two phases. At concentrations less than 2.5 M, the two-phase aqueous system does not shape. At low concentrations, salts like sodium chloride, potassium iodide, and potassium chloride did not form a two-phase structure. Due to its high solubility, ammonium sulfate was chosen as the salt-forming phase.

### Impact of initial cobalt solution volume

**Figure 6:**
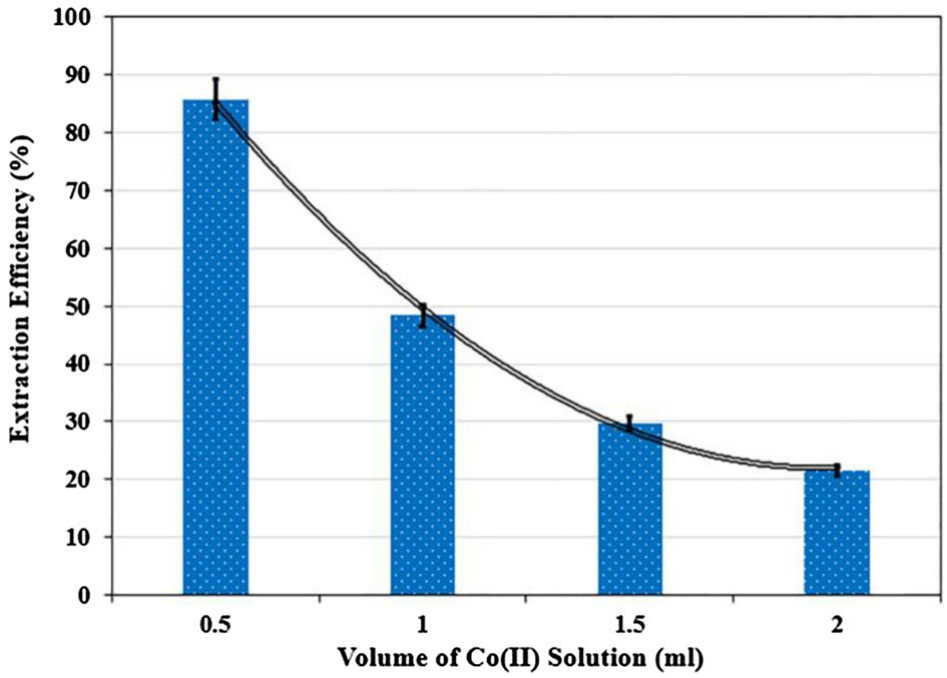
Effect of volume of cobalt solution on the extraction of Co(II) (T = 25 °C, Time = 30 min, PEG = 50% w/w, (NH_4_)_2_SO_4_ = 4 M, pH = 2).

The impact of initial cobalt solution volume (500 ppm) in a two-phase system of 50% (w/w) polyethylene glycol (3 ml) /4 M ammonium sulfate solution (3 ml) in the range of 0.5–2 ml was studied. The results obtained in Fig. 6 showed that with increasing cobalt solution volume, the extraction percentage decreases. Increasing the metal content shifted the equilibrium toward the formation of the [M(SO_4_)_x_]^(2x−m)-^ metal complex, favoring the fraction of species containing the metal. This behavior was not observed for cobalt ions because K[Co(SO_4_)_2_]^2−^ was the highest, favoring the formation of a greater concentration of the complexes, even at low cobalt concentration.

### Effect of the (PEG/ (NH_4_)_2_SO_4_) phase ratio

The effect of the phase ratio of polyethylene glycol to ammonium sulfate on extraction efficiency is shown in Table 2. As the results show, increasing the volume ratio of phases from 0.75 to 2 ml improves cobalt extraction. In two-phase systems, this may be due to significant concentration variations. In other words, as polyethylene glycol concentrations increase, the hydrogen bond between polyethylene glycol molecules and water strengthens, expanding the interface between the two phases^48^. According to Table 2, the extraction efficiency is little influenced by the changes in the volume ratio between phases. The distribution coefficients of complex species are sufficiently high to produce a quantitative extraction even with a lower volume of polymer-rich phase.

**Table 2:** Effect of phase ratio on the extraction of Co(II) (T = 25 °C; Time = 15 min; Co(II) = 500 ppm, PEG = 50% w/w, pH = 2).

Phase ratio (PEG solution/(NH_4_)_2_SO_4_ solution)	Extraction efficiency (%)
0.75	86.65 ± 0.27
1	86.81 ± 0.13
1.25	87.05 ± 0.23
1.5	87.63 ± 0.21
2	88.34 ± 0.32

### Effect of the aqueous pH

**Figure 7:**
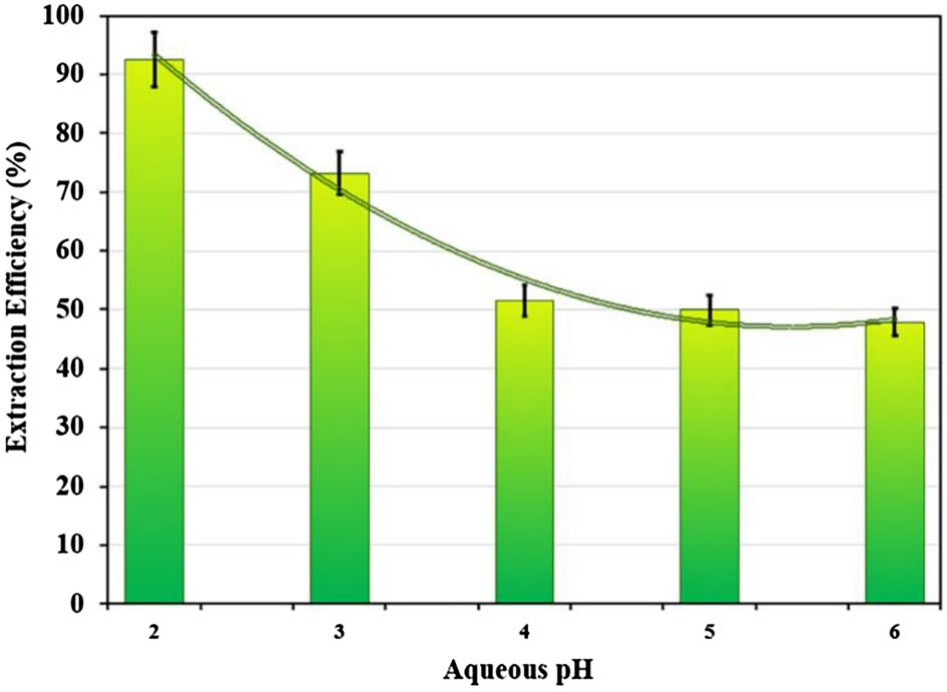
Effect of pH on the extraction of Co(II) (T = 25 °C, Time = 30 min, Co(II) = 500 ppm, PEG = 50% w/w, (NH_4_)_2_SO_4_ = 4 M).

The effect of pH of saline solution on the percentage of cobalt extraction was investigated in the pH range of 2–7. The extraction percentage decreases with increasing pH, as shown in Fig. 7. This decline in extraction percentage may be due to cobalt deposition at high pH. In addition, raising the pH of the salt lowers the hydrophobicity of polyethylene glycol, improving extraction efficiency^49^. Formation of the Co(SO_4_)_2_^2−^ complexes is observed in acidic or neutral solution. The high value of pH led to the formation of hydroxide complexes, which could interfere with the interactions between the metal complexes and the macromolecule. Also, the reduction in the pH of the saline solution led to the increment in the hydrophobicity of the PEG-rich phase and, thus, enhancement of the efficiency of metal extraction decreases with the reduction acidity of the saline solution^49,50^. The result of pH value equal to 2 was determined to be the optimum pH based on the data and it is the main factor in controlling the kinetic of cobalt extraction.

The CoSO_4_ species added in the saline solution are pushed to the interface due to their incompatibility with the high hydrated environment of this phase. At the interface, the CoSO_4_ species will interact with one supplementary sulfate ions and form the anionic complexes of CO(SO_4_)_2_^2−^. The hydration degree of stable anionic species is lower enough to be compatible with the low hydrated environment of PEG-rich phase. Therefore, the transfer of complex was carried out to PEG-rich.

### Effect of extraction time

**Figure 8:**
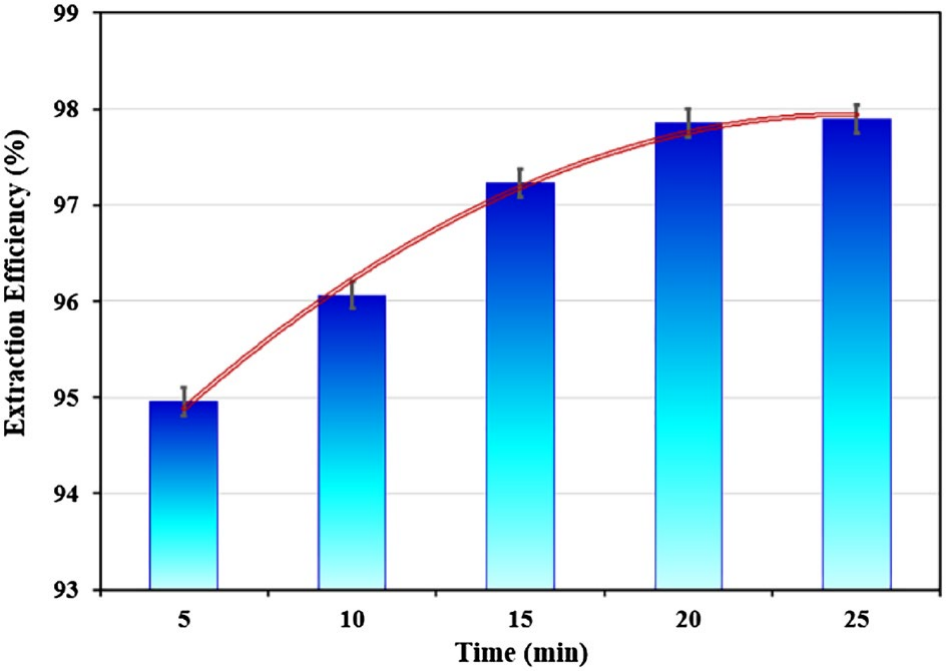
Effect of time on the extraction of Co(II) (T = 25 °C, Co(II) = 500 ppm, PEG = 50% w/w, (NH_4_)_2_SO_4_ = 4 M, pH = 2).

The effect of time on the extraction efficiency of polyethylene glycol 1000/ammonium sulfate was investigated. The extraction efficiency results for times 5, 10, 15, 20, and 25 min are shown in Fig. 8. The results show that extraction efficiency increases over time. As a result, the best value was observed to be 15 min.

### Effect of extraction temperature

**Figure 9:**
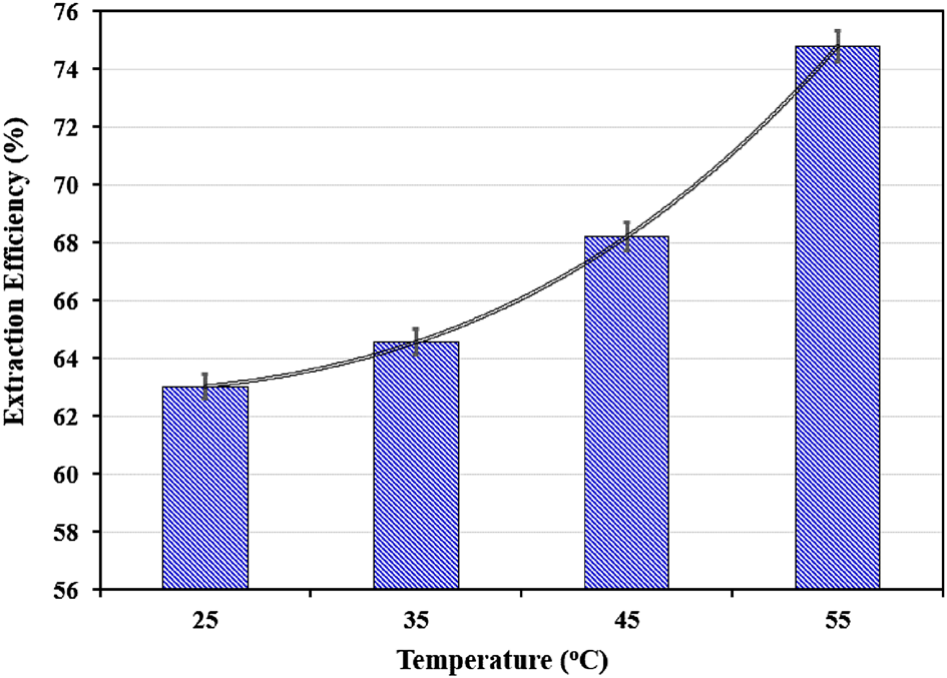
Effect of temperature on the extraction of Co(II) (Time = 30 min, Co(II) = 500 ppm, PEG = 50% w/w, (NH_4_)_2_SO_4_ = 4 M, pH = 2).

The extraction efficiency of cobalt was studied at different temperatures 25–55 °C. As the results of Fig. 9 showed that increasing the temperature leads to increasing the extraction efficiency. To put it another way, as the temperature rises, the biphasic area expands. It has been proved that two phase regions in aqueous two phase systems will expand at higher temperatures^51^.

### PEG-Co (II) vibrational spectroscopic analysis

**Figure 10:**
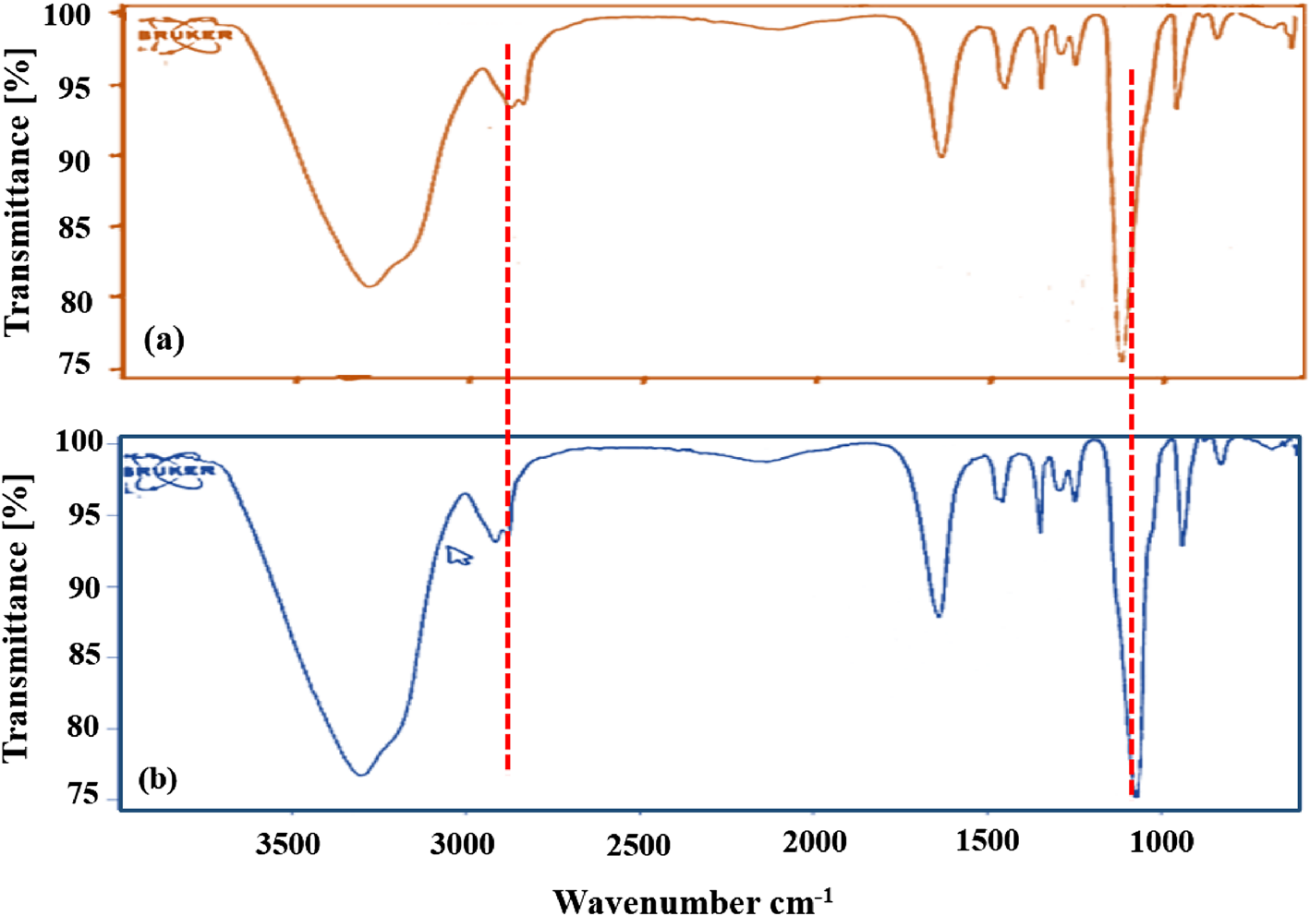
IR spectra of PEG and PEG-Co(II).

Figure 10 shows the infrared spectrum of polyethylene glycol and polyethylene glycol-cobalt before (Fig. 10a) and after extraction (Fig. 10b). As the figures appeared, the absorption peak at 3450 cm^−1^ is identified with the tractable vibration of the O–H group in polyethylene glycol. It tends to be considered that to be cobalt ions was added to the PEG, the absorption peak of the C–O–C bond in the 1060–1100 (cm^−1^) span movements to a low wavenumber.

This showed that the asymmetric stretching vibration of the C–O–C bond in this area was diminished by the vibration frequency because of the impact of Co(II). It tends to be construed that Co(II) fundamentally shaped a complex structure with oxygen atoms in the polyethylene glycol. The absorption peak of methylene (2850–2900) shifts from 50 to 60 cm^−1^ with the presence of Co(II). The strengthening of the C-H bond moves the absorption peak to a higher wavenumber after the coordination of PEG with Co(II).

### Thermo-gravimetric analysis

**Figure 11:**
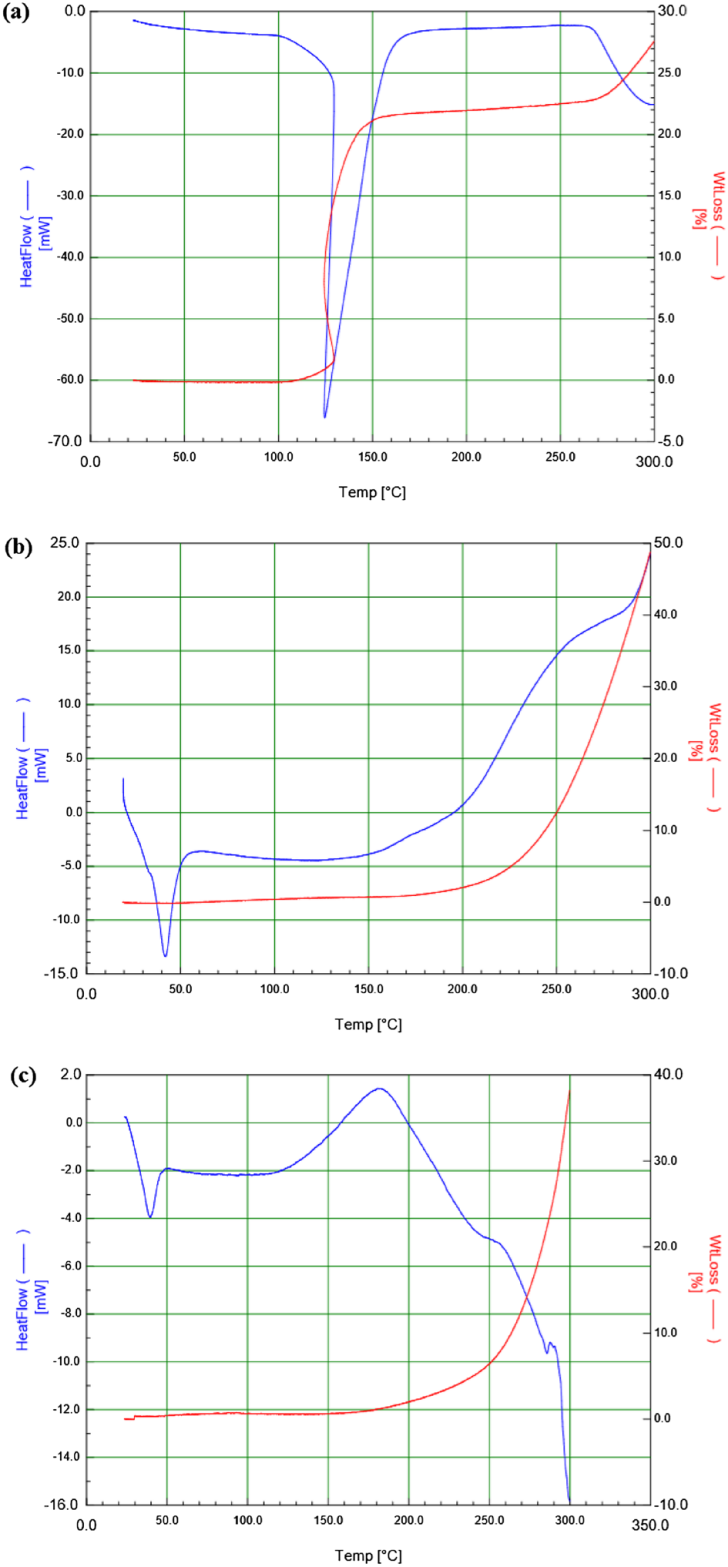
TG and DTG Curves of PEG, Co(II) and PEG-Co(II).

The thermo-gravimetric examination of PEG1000, cobalt salt and PEG-Co(II) are appeared in Fig. 11. The PEG1000, and cobalt salt disintegrated at 225 °C and 135 °C, individually. The thermal stability of PEG1000-Co(II) complex isn't affected in the analyses and the underlying disintegration temperature of PEG-Co(II) was 225 °C. The decomposition temperature of PEG-Co(III) was higher than that of cobalt salt, and the melting point of PEG-Co(II) was higher than that cobalt salt. The coordination response among PEG and Co(II) happens rather than straightforward actual extraction.

### Extraction of other heavy metals

**Figure 12:**
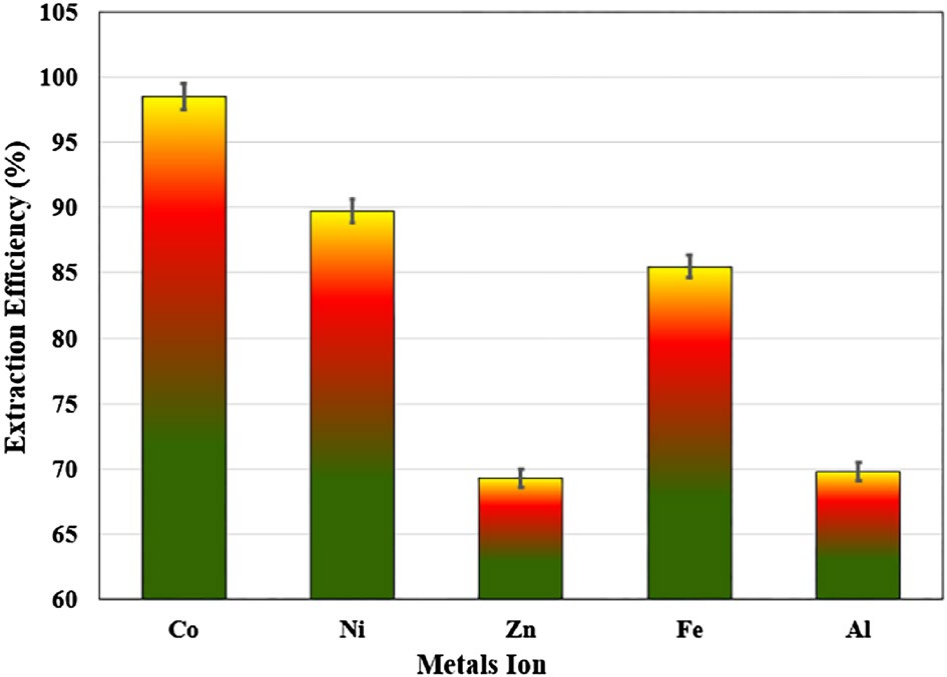
Extraction of other heavy metal ions with PEG1000-ammonium sulfate system.

The two-phase aqueous system (PEG + (NH_4_)_2_SO_4_) was also investigated to extract other metal ions. The simulated wastewater from the leaching solution of cobalt cake from zinc plant residue containing nickel ~ 0.054 g/L, cobalt ~ 1.523 g/L, zinc ~ 0.976 g/L, iron ~ 0.250 g/L and aluminum ~ 0.138 g/L ions (aqueous pH = 2) was investigated in the second step. The obtained results are shown in Fig. 12.

The concentration of cobalt ions in the effluent reached 0.024 g/L at the highest PEG1000 with 50% (w/w) and 55 °C (%E ~ 98.49). The nickel ions were also efficiently extracted with the trace level of 0.0055 g/L in the effluent (%E ~ 89.76). The extraction efficiency of other metals (Fe, Al, Zn) in PEG-rich phase is obtained equal to 69.24%, 69.74%, 69.26%, respectively. The selectivity of cobalt ions to other metals (β_Co/M_) was obtained equal to 7.47, 29.08, 11.13, 28.44 for nickel, zinc, iron, and aluminum. Thus, the PEG1000-(NH_4_)_2_SO_4_ effectively extracted cobalt ions and other heavy metals, and showed excellent application potential for treating of real wastewater.

## Conclusion

This research used a two-phase aqueous system to extract cobalt, with ammonium sulfate as the salt-forming phase and polyethylene glycol 1000 as the polymer-forming phase. The effects of salt concentration, polyethylene glycol concentration, phasing ratio, aqueous pH, and extraction time were investigated on the extraction of cobalt ions. The results showed that increasing the concentration of polyethylene glycol and salt, the volume ratio of phases and extraction time increases extraction efficiency, while increasing pH and initial metal concentration decreases extraction efficiency. Also, in this study, salts such as potassium chloride, potassium fluoride, potassium iodide were used that the two-phase aqueous system was not formed in concentrations below 30%. Extraction efficiency was achieved above 98% without the use of an extractant. The evaluated system in this study is a promising strategy without the application of toxic extractants for cobalt recovery from wastewater.

## Data Availability

The datasets used and/or analyzed during the current study are available from the corresponding author on reasonable request.
